# Description of an Australian endemic species of *Trioza* (Hemiptera: Triozidae) pest of the endemic tea tree, *Melaleuca alternifolia* (Myrtaceae)

**DOI:** 10.1371/journal.pone.0257031

**Published:** 2021-09-22

**Authors:** Francesco Martoni, Mark J. Blacket

**Affiliations:** Agriculture Victoria Research, AgriBio Centre, Bundoora, Victoria, Australia; University of Saskatchewan College of Agriculture and Bioresources, CANADA

## Abstract

Psyllids, also known as jumping plant lice, are phloem feeding Hemiptera that often show a strict species-specific relationship with their host plants. When psyllid-plant associations involve economically important crops, this may lead to the recognition of a psyllid species as an agricultural or horticultural pest. The Australian endemic tea tree, *Melaleuca alternifolia* (Maiden & Betche) Cheel., has been used for more than a century to extract essential oils and, long before that, as a traditional medicine by Indigenous Australian people. Recently, a triozid species has been found to damage the new growth of tea trees both in Queensland and New South Wales, raising interest around this previously undocumented pest. Furthermore, adults of the same species were also collected from *Citrus* plantations, leading to potential false-positive records of the exotic pest *Trioza erytreae* (Del Guercio 1918), the African Citrus psyllid. Here we describe for the first time *Trioza melaleucae* Martoni **sp. nov.** providing information on its distribution, host plant associations and phylogenetic relationships to other *Trioza* species. This work enables both morphological and molecular identification of this new species, allowing it to be recognized and distinguished for the first time from exotic pests as well as other Australian native psyllids. Furthermore, the haplotype network analysis presented here suggests a close relationship between *Trioza melaleucae* and the other Myrtaceae-feeding *Trioza* spp. from Australia, New Zealand, and Taiwan.

## 1. Introduction

The Australian psyllid fauna includes more than 400 species [1, 2] belonging to six of the seven families of Psylloidea worldwide [3]. While the vast majority of Australian native psyllids belong to the families Aphalaridae (i.e., more than 130 species within *Glycaspis;* [1]) and Psyllidae (i.e., 40 described and more than 60 undescribed species within *Acizzia*; [4]), the family Triozidae has been attracting the interest of entomologists and taxonomists during the last 20 years. A number of endemic genera have been described recently, including *Myotrioza* [5], *Acanthocasuarina* [6], and *Casuarinicola* [7], while adventive species have been recorded for the first time for Australia, such as *Bactericera cockerelli* (Šulc), the tomato potato psyllid, recently reported near Perth in Western Australia [8].

Today, in Australia, the family Triozidae includes more than 70 species, both native and adventive, belonging to a total of 10 genera [1, 2, 9]. Within the Triozidae, the genus *Trioza* includes 11 described species [1, 10–12]. Of these, three are known to be hosted by Myrtaceae: *Trioza adventicia* Tuthill and *T*. *eugeniae* Froggatt [12], on *Syzygium* P. Browne ex Gaertn., and *T*. *tristaniae* Froggatt, on *Lophostemon* Schott. No *Trioza* species have been recorded in the literature associated with the plant genus *Melaleuca* L. (Myrtaceae); however, recently an undetermined triozid psylloid was found to be damaging tea tree plants in eastern Australia [13].

The presence of a psyllid on *Melaleuca* has horticultural and economic implications, as the Australian endemic tea tree, *Melaleuca alternifolia* (Maiden & Betche) Cheel (Myrtaceae), has strong economic and cultural importance. Culturally, the group of “tea trees”, of which *M*. *alternifolia* is the most common and most used example, has been utilised by Indigenous Australians of eastern inland areas as a traditional medicine [14]. Crushed leaves produce oils that have been used to treat coughs and colds via inhalation, or sore throat via infusion. Furthermore, leaves were applied on wounds and to treat skin ailments [14]. Indeed, antiseptic and anti-inflammatory properties, as well as antimicrobial ones, have been demonstrated and widely documented [15]. Economically, Australian production of tea tree oil is currently estimated at 900 tonnes, with a value of AUD 35.32 million, with around 4,000 hectares under tea tree production in Australia [16].

Here we present the description of the tea tree triozid, *Trioza melaleucae* sp. nov., together with the first data on its ecology, host plant association and phylogenetic relationships. This provides the first fundamental information for further study on this species and its relationship with a horticulturally important plant.

## 2. Materials and methods

### 2.1. samples

Samples of an undescribed *Trioza* sp. were collected between September and December 2020 from two locations in Australia. The first population was sampled from a *Citrus* plantation (on boundary trees adjacent to a tea tree plantation) near Dimbulah, in Queensland, while a second population was sampled from a tea tree plantation near Teven, in New South Wales. Holotypes and paratypes were deposited in the Victorian Agriculture Insect Collection (**VAIC**), in Melbourne (Victoria, Australia), while additional paratypes were deposited at the Australian National Insect Collection (**ANIC**), in Canberra (Australian Capital Territory, Australia). High resolution photos of the syntype of *Trioza tristaniae* Froggatt (a single wing) were provided by ANIC.

### 2.2. Specimen preparation, measurements, drawings, and photographs

Microscope slide preparation, following the work of Taylor *et al*. [5], was performed on eight specimens: six from the New South Wales population (three males and three females), and two from the Queensland population (a male and a female). Morphology of adult characters follows the work of Rendón-Mera *et al*. [17]. High-resolution automontage photographs of adults and nymphs were obtained using the Leica Application Suite software (version 4.5.0), from five to 20 stacked images obtained using a Leica stereo microscope M205C with a DFC450 camera. Measurements were obtained using the Leica ‘Segment Line Tool’ from four males and four females. High resolution photos where then collated into plates using the GNU Image Manipulation Program (GIMP) version.2.10.20. The line drawings were made using the software Inkscape v.0.92.3. from fresh specimens, high resolution photos or other illustrations.

### 2.3. Molecular analysis

#### 2.3.1. DNA extraction, amplification, and sequencing

DNA was non-destructively extracted from a total of eight single psyllids (four males and four females) using the protocol presented elsewhere for Muscidae [18]. A fragment of the subunit I of the *Cytochrome Oxidase* gene (COI) barcode region [19] of approximately 570 bp was targeted using the primers PsyCOI-F3 (5’-ACAATTGTTACWGCWCAYGC-3’; [20]) and HCO2198 (5’-TAAACTTCAGGGTGACCAAAAAATCA-3’; [21]). The polymerase chain reaction (PCR) was performed using the MyFi kit (Bioline Meridian Biosciences, Cincinnati, USA) following the manufacturer’s instructions and the following cycle: initial denaturation at 95 °C for 5 mins, followed by 35 cycles of 30 s at 94 °C, 30 s at 50 °C and 1 min at 72 °C, and a final elongation of 7 min at 72 °C. PCR products were Sanger sequenced in both directions commercially (Macrogen, Seoul, Korea). The electropherograms were manually examined and checked for pseudogenes and stop codons using the software MEGA X [22]. Forward and reverse sequences were combined in MEGA X and each sequence was blasted against the online database GenBank and BOLD, to assess similarities to other taxa.

#### 2.3.2. COI genetic distance

The eight COI sequences generated here were aligned with those of *Trioza curta* (Ferris & Klyver) and *T*. *adventicia* since they were found to be the closest matches on GenBank (see below). A pairwise distance matrix using the Kimura-2-parameters (K2P; [23]) model was generated using MEGA X to assess genetic distances both between the two sampled populations of *T*. *melaleucae*, and between these and other most closely related triozid species available on GenBank.

#### 2.3.3. COI Haplotype network analysis

The COI sequences obtained in this study were trimmed to be aligned together with the shortest sequences (194 bp) included in the dataset presented by Taylor and Martoni [12], in order to compare them with the only DNA sequences available of *T*. *eugeniae* Froggatt. Sequences and accession numbers used are reported in S1 Table. The software PopART [24] was used to perform a Median Joining Network analysis [25] with ε = 0.

### 2.4. Nomenclatural act

The electronic edition of this article conforms to the requirements of the amended International Code of Zoological Nomenclature (ICZN) [26, 27], and hence the new name contained herein is available under that Code from the electronic edition of this article. This published work and the nomenclatural act it contains have been registered in ZooBank, the online registration system for the ICZN. The ZooBank LSIDs (Life Science Identifiers) can be resolved and the associated information viewed through any standard web browser by appending the LSID to the prefix “http://zoobank.org/”. The LSID for this publication is: urn:lsid:zoobank.org:pub:9D340AFC-E8F6-4CCD-BCE5-B310DFBEE16E. The electronic edition of this work was published in a journal with an ISSN, and has been archived and is available from the following digital repositories: PubMed Central, LOCKSS

## 3. Results

### 3.1. Taxonomy

#### 3.1.1. *Trioza melaleucae* Martoni, 2021

urn:lsid:zoobank.org:act:4959158A-2548-4F68-BFE4-72A8651A38A3.

**Fig 1:**
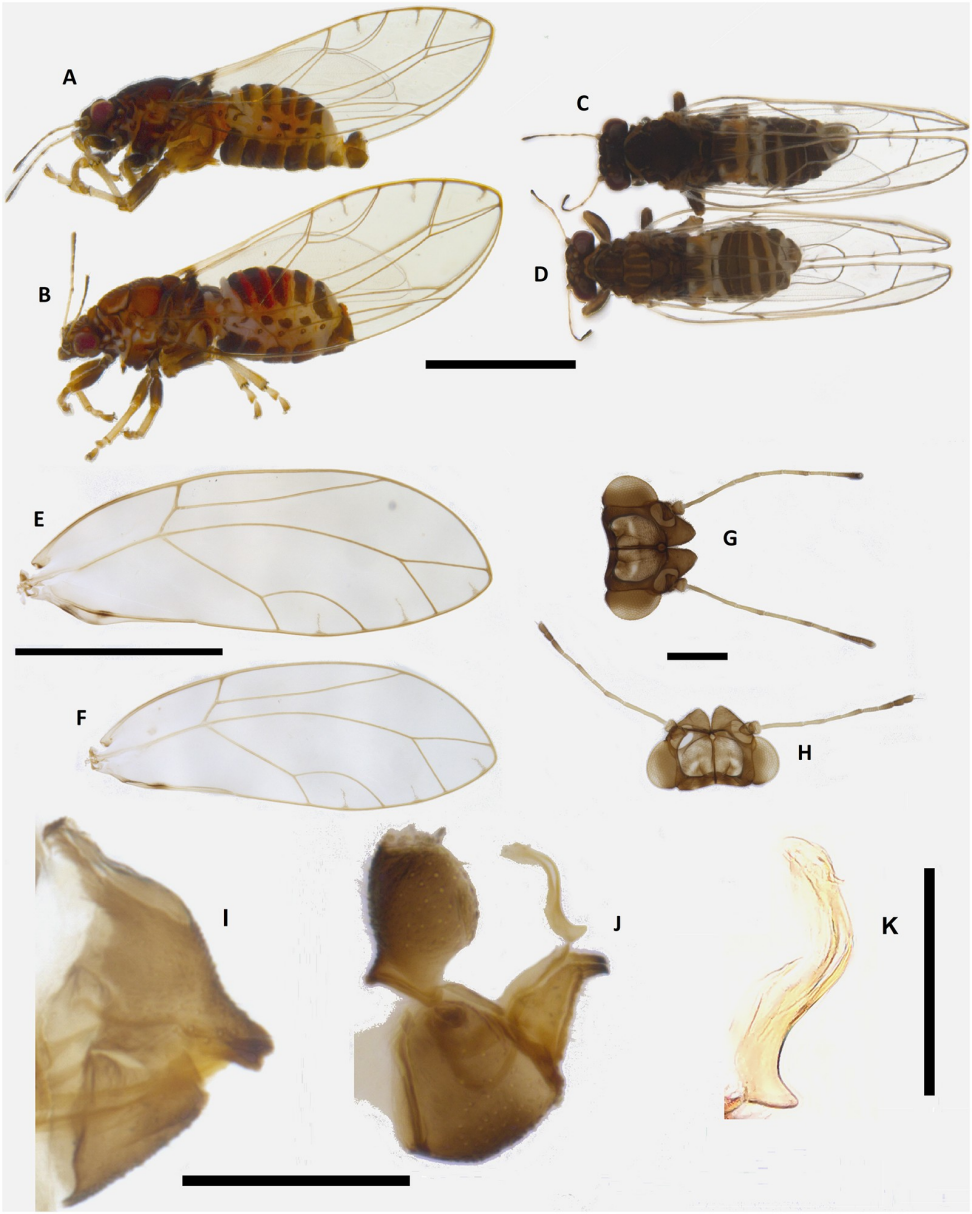
*Trioza melaleucae*. Habitus lateral of male (**A**) and female (**B**); habitus dorsal of male (**C**) and female (**D**), wings of female (**E**) and male (**F**), head of female (**G**) and male (**H**), terminalia of female (**I**) and male (**J**), with male aedeagus (**K**). Scale bars are 1 mm (A-F), 0.2 mm (G-J) and 0.1mm (K).

**Fig 2:**
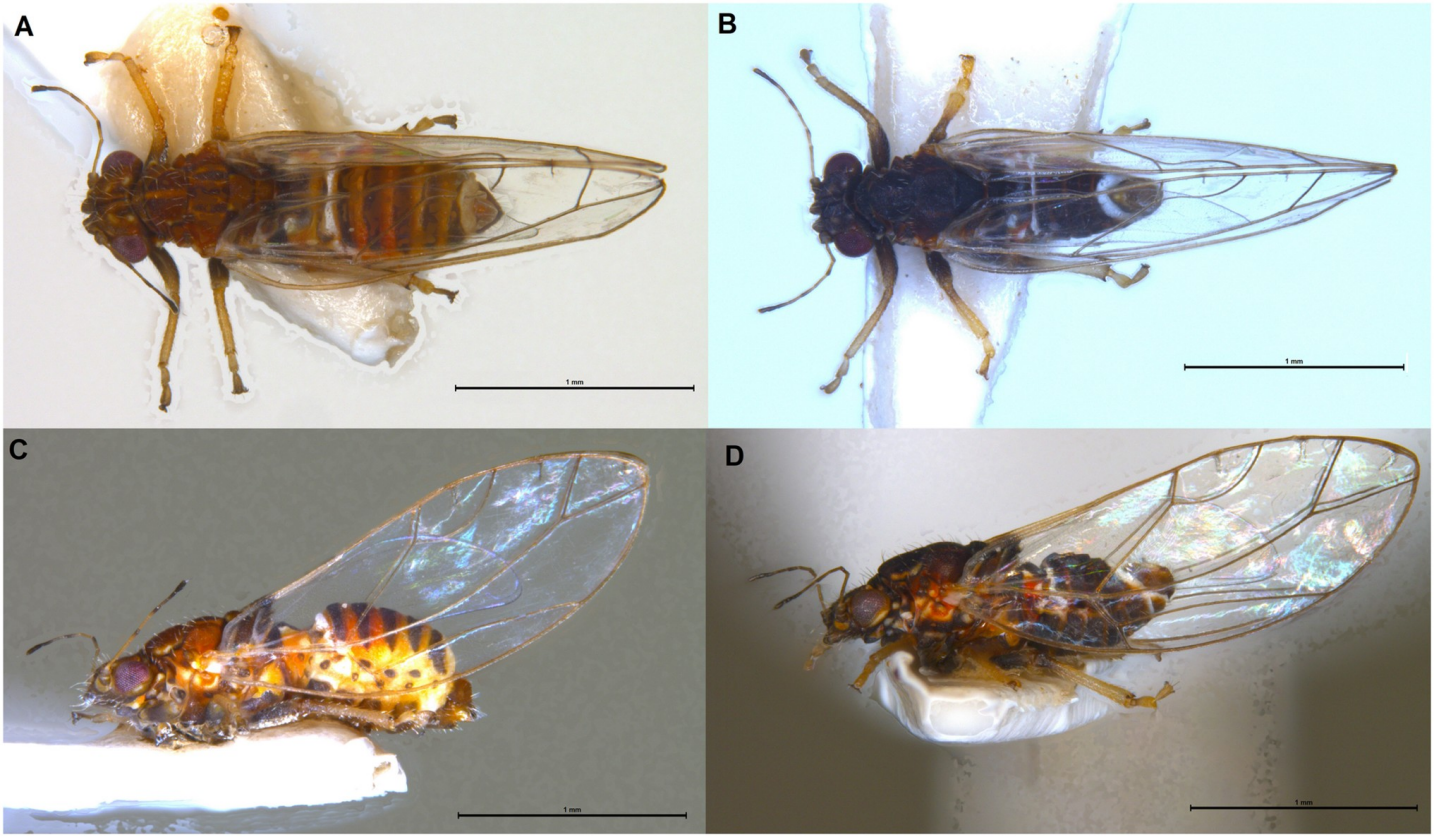
Adults of *Trioza melaleucae*. Habitus dorsal of female (**A**) and male (**B**); habitus lateral of female (**C**) and male (**D**). The scale bars are 1 mm.

**Fig 3:**
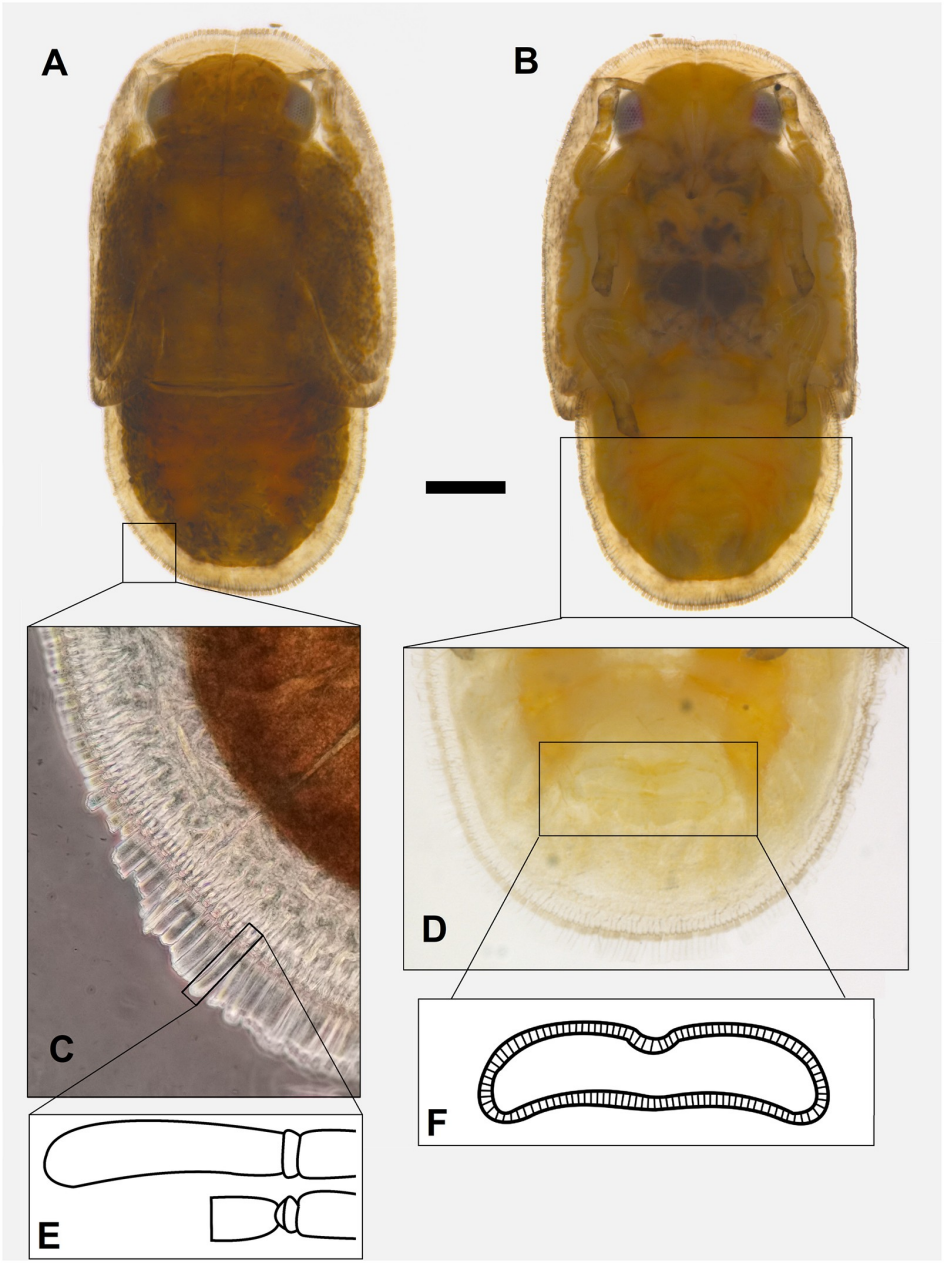
*Trioza melaleucae* nymphs. Habitus dorsal (**A**), habitus ventral (**B**) and particulars of the sectasetae **(C)—**showing the variation in length due to presence/absence of the filamentous waxy exudate **(E)**—and of the anal opening (**D, F**). The scale bars is 0.2 mm.

Figs 1 – 3, 5A, 6A, 7A.

#### 3.1.2. Material examined

*3*.*1*.*2*.*1*. *Holotype*. (Male) deposited at the **VAIC**. Entire specimen mounted on card triangle. Labels: ‘Australia, NSW / Teven / 28°50’51.5"S 153°29’10.5"E / Nov 2020 P. Entwistle / On *Melaleuca alternifolia*’ {Printed on white card}. ‘HOLOTYPE ♂ / *Trioza melaleucae* / Martoni 2021’ {Printed on red card}.

*3*.*1*.*2*.*2*. *Paratypes*. 1 ♀, 1 ♂ deposited at the **VAIC**. Entire specimen mounted on card triangle. Labels: ‘Australia, NSW / Teven / 28°50’51.5"S 153°29’10.5"E / Nov 2020 P. Entwistle / On *Melaleuca alternifolia*’ {Printed on white card}. ‘PARATYPE ♂-♀ / *Trioza melaleucae* / Martoni 2021’ {Printed on blue card}; 2 ♀, 2 ♂ deposited at the **ANIC**. Entire specimen mounted on card triangle. Labels: ‘Australia, NSW / Teven / 28°50’51.5"S 153°29’10.5"E / Nov 2020 P. Entwistle / On *Melaleuca alternifolia*’ {Printed on white card}. ‘PARATYPE ♂-♀ / *Trioza melaleucae* / Martoni 2021’ {Printed on blue card}; 1 ♀, 1♂ deposited at the **VAIC**. Dissected specimen mounted on microscope slide. Labels: ‘Australia, NSW / Teven / 28°50’51.5"S 153°29’10.5"E / Nov 2020 P. Entwistle / On *Melaleuca alternifolia*’ {Printed on white card}. ‘PARATYPE ♂-♀ / *Trioza melaleucae* / Martoni 2021’ {Printed on blue card}. 1 ♀, 1♂ deposited at the **VAIC**. Dissected specimen mounted on microscope slide. Labels: ‘Australia, QLD / Dimbulah / Nov 2020 S. Stow / On *Melaleuca alternifolia*’ {Printed on white card}. ‘PARATYPE ♂- ♀/ *Trioza melaleucae* / Martoni 2021’ {Printed on blue card}.

*3*.*1*.*2*.*3*. *Other samples examined for this study*. A few hundred specimens from the populations listed above in addition to photographic material from the population listed in the “distribution” section, below.

#### 3.1.3. Diagnosis

*Trioza melaleucae* belongs to the group of Myrtaceae-feeding *Trioza* present in Australia and New Zealand, as well as other parts of the Austro-Pacific. Australian and New Zealand members include *T*. *adventicia*, *T*. *eugeniae*, *T*. *tristaniae* and *T*. *curta*. Diagnosis of these species can be made easily based on geographic distribution and host plant association, if this information is available (Table 1). *Trioza curta* is endemic to New Zealand and hosted by *Metrosideros* Banks ex Gaertn.; *T*. *adventicia* is native to Australia (but present also in New Zealand and USA) and hosted by *Syzygium*; while *T*. *eugeniae* and *T*. *tristaniae* are both endemic to Australia, but hosted by *Syzygium* and *Lophostemon confertus* (R.Br.) Peter G.Wilson & J.T.Waterh., respectively [12, 28]. Furthermore, *T*. *tristaniae* differs from the other species listed here in being a gall-forming psyllid, instead of a pit-forming one [28]. Hence, *T*. *melaleucae* is the only known pit-forming species endemic to Australia and hosted by *Melaleuca* (Table 1). When relying only on morphology, the size of the body and of the wings of *T*. *melaleucae* is smaller than that of all other species (Table 1).

**Table 1:** Distributional, ecological and morphological data from the six *Trioza* species compared in this study.

	*T*. *melaleucae*	*T*. *curta*	*T*. *adventicia*	*T*. *eugeniae*	*T*. *tristaniae*	*T*. *erytreae*
**Origin**	Australia	New Zealand	Australia	Australia	Australia	Africa
**Additional distribution**	Endemic	Endemic	New Zealand, USA	Endemic	Endemic	Portugal, Spain
**Host plant**	*Melaleuca*	*Metrosideros*	*Syzygium*	*Syzygium*	*Lophostemon*	*Citrus*
	(Myrtaceae)	(Myrtaceae)	(Myrtaceae)	(Myrtaceae)	(Myrtaceae)	(Rutaceae)
**Ecology**	Pit-former	Pit-former	Pit-former	Pit-former	Gall-former	Pit-former
**Body length (genal processes to apex of wings)**	♂ 2.5 ♀ 3.0	♂ 3.05 ♀ 3.4	♂ 3.2 ♀ 3.57	♂ 3.2 ♀ 3.7	N/A	♂3.55 ♀ 3.85
**Wing length**	♂2.2 ♀2.2	♂2.66 ♀2.92	♂3.0 ♀3.3	♂2.9 ♀3.1	♂/♀3.0	♂2.6 ♀3.2
**Value cell cu1**	♂2.36 ♀2.24	♂2.21 ♀2.22	♂2.35 ♀2.38	♂2.35 ♀2.44	♂/♀1.21	♂2.7 ♀2.72
**Value cell m1**	♂1.24 ♀1.22	♂1.35 ♀1.38	♂1.15 ♀1.14	♂1.24 ♀1.24	♂/♀1.29	♂1.09 ♀1.22

**Fig 4:**
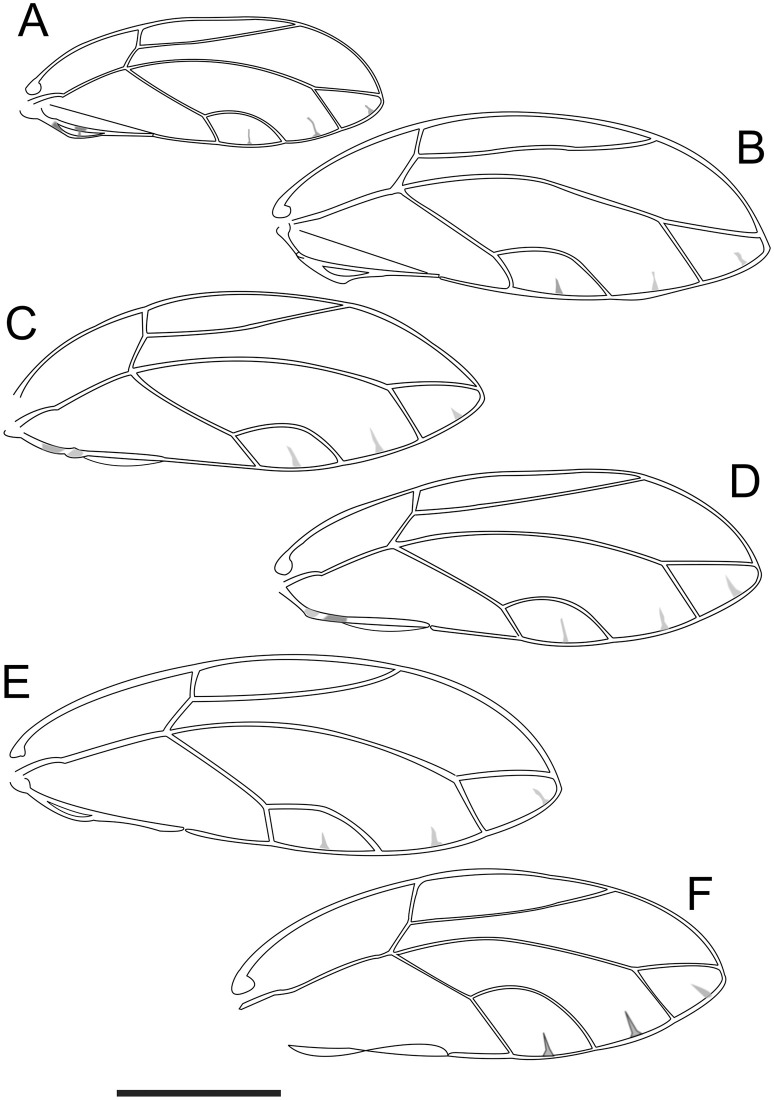
Female wings of *Trioza melaleucae* (**A**), *T*. *curta* (**B**), *T*. *adventicia* (**C**), *T*. *eugeniae* (**D**), *T*. *erytreae* (**E**) and *T*. *tristaniae* (**F**). Scale bar is 1 mm.

**Fig 5:**
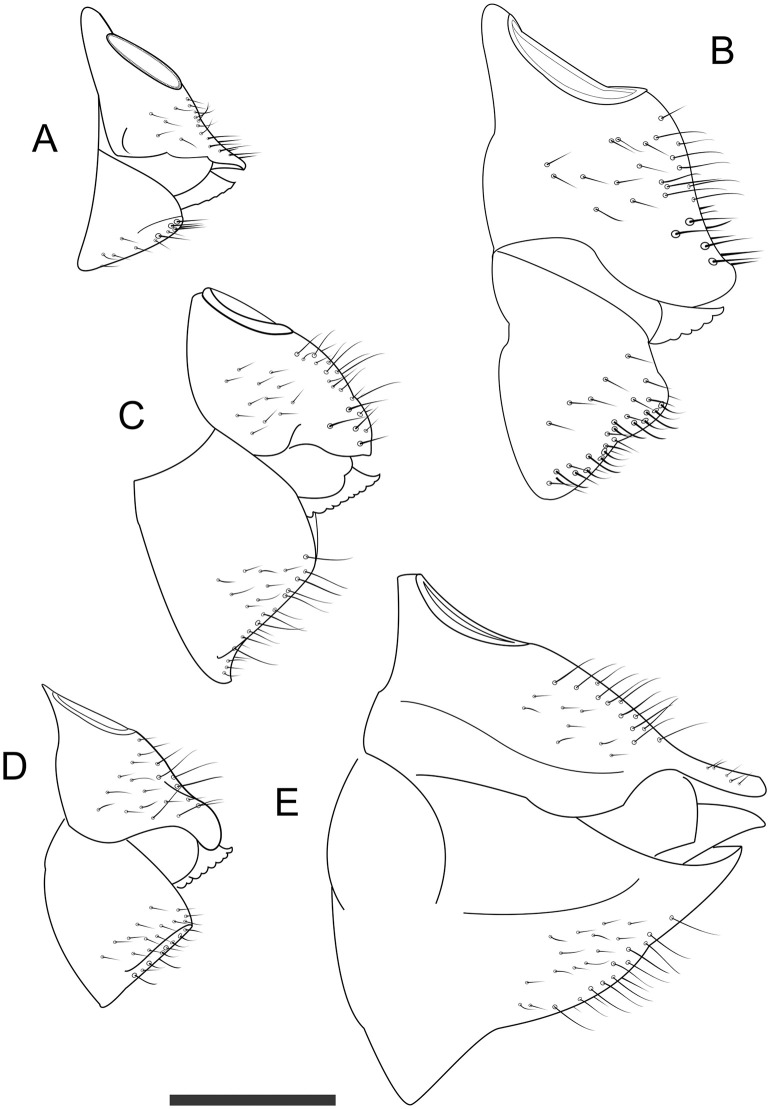
Lateral view of female terminalia of *Trioza melaleucae* (**A**), *T*. *curta* (**B**), *T*. *adventicia* (**C**), *T*. *eugeniae* (**D**) and *T*. *erytreae* (**E**). Scale bar is 0.2 mm.

The general body shape of *T*. *melaleucae* is similar to that of *T*. *curta*, but the two species can be distinguished based on dimensions, wings markings and shape of female terminalia. *T*. *melaleucae* is smaller, with its overall dimensions barely reaching 3 mm for females, with males about 2.5 mm, while the male of *T*. *curta* can reach 3.30 mm, with the wings alone reaching 2.8 mm for males and 3.10 mm for females [29], against the wings of *T*. *melaleucae* which are always shorter than 2.4 mm (Fig 4). The wings of *T*. *melaleucae* present a distinctive marking on the A vein, around the point where this widens to create cell a1, while this marking is lacking in *T*. *curta*. Vein Rs is shorter in *T*. *melaleucae* than in *T*. *curta* (Fig 4). The female terminalia, when observed laterally, are more slender than those of *T*. *curta* (Fig 5), with the ratio between proctiger length and head width being only 0.38–0.47, against the ratio of 0.5–0.55 in *T*. *curta*, and not showing a post-anal bump (present in *T*. *curta*) (Fig 5 [29]).

**Fig 6:**
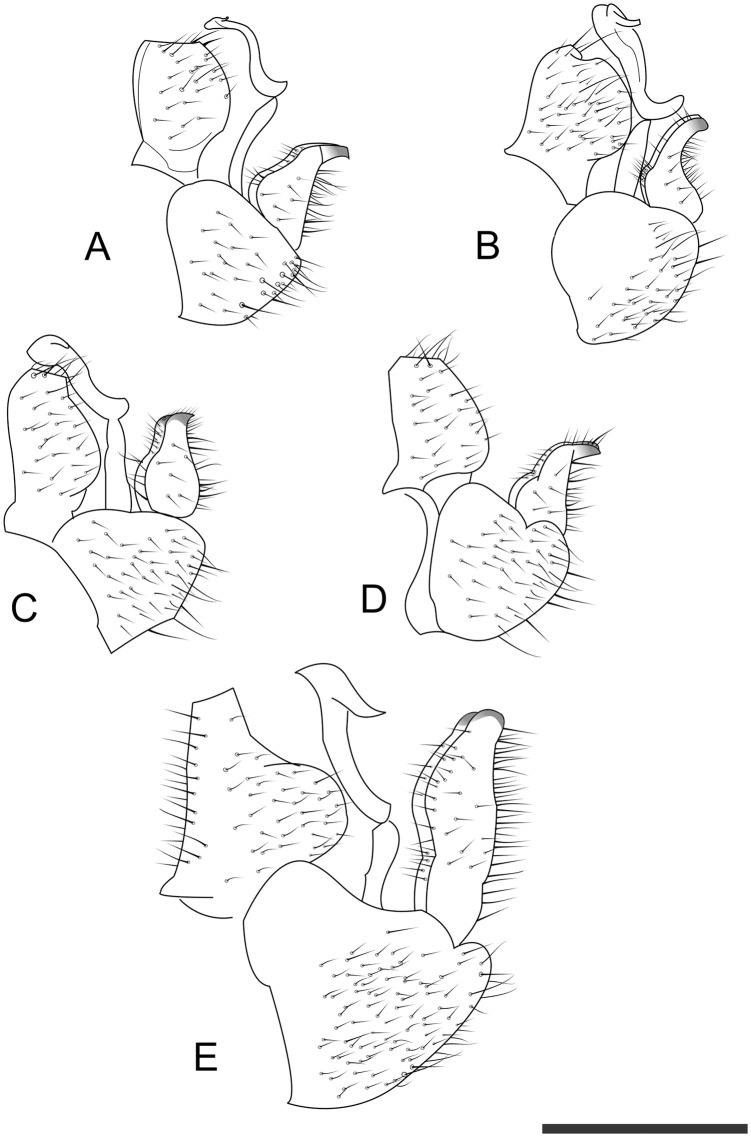
Lateral view of male terminalia of *Trioza melaleucae* (**A**), *T*. *curta* (**B**), *T*. *adventicia* (**C**), *T*. *eugeniae* (**D**) and *T*. *erytreae* (**E**). Scale bar is 0.2 mm.

**Fig 7:**
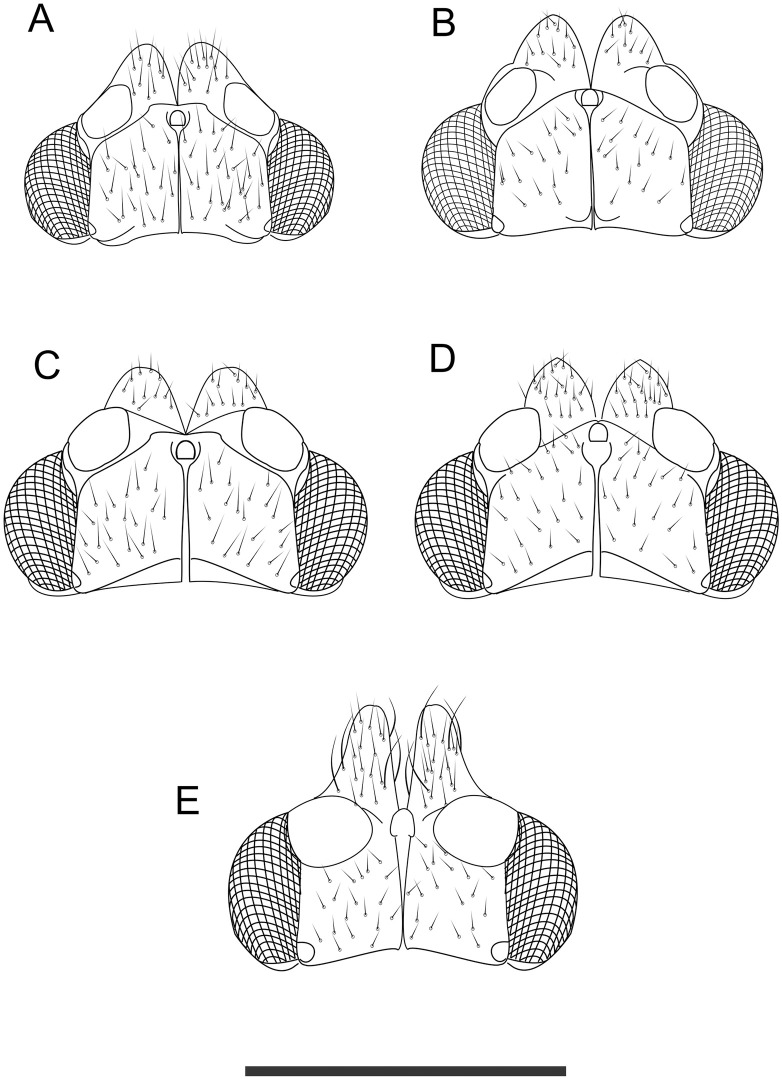
Dorsal view of head of *Trioza melaleucae* (**A**), *T*. *curta* (**B**), *T*. *adventicia* (**C**), *T*. *eugeniae* (**D**) and *T*. *erytreae* (**E**). Scale bar is 0.2 mm.

When compared to *T*. *adventicia* and *T*. *eugeniae*, *T*. *melaleucae* remains easily distinguishable based on its smaller size (Table 1). While the wings of all three species share a very similar marking on the veins around cell a1 (Fig 4), the wings of *T*. *melaleucae* remain noticeably smaller (♂ 2.09–2.36, ♀ 1.98–2.35) than those of *T*. *adventicia* (♂ 2.90–3.10, ♀ 3.24–3.41) and *T*. *eugeniae* (♂ 2.72–3.07, ♀ 3.03–3.17) [12]. The wing apex is also rounder in shape (Fig 5). Additionally, the terminalia of both sexes are quite distinctive. In lateral view, the female terminalia of *T*. *melaleucae* appear more slender (Fig 5A), without the post-anal bump present in both *T*. *adventicia* and *T*. *eugeniae* (Fig 5C and 5D). Male terminalia of *T*. *melaleucae* (Fig 6A), in lateral view, are more similar to those of *T*. *eugeniae* (Fig 6D), with parameres more slender and thinner in apical half than those of *T*. *adventicia* (Fig 6C). However, in lateral view, the general shape of the proctiger is shorter and rounder in *T*. *melaleucae* (Fig 6A) than it is in both *T*. *adventicia* (Fig 6C) and *T*. *eugeniae* (Fig 6D). In lateral view, the subgenital plate of *T*. *melaleucae* has a shorter base, contributing to a less rounded, more triangular shape than that of *T*. *eugeniae*. Additionally, the proctiger appears to be of a rounder shape than that *of T*. *eugeniae*. From a dorsal point of view, the genal processes of *T*. *melaleucae* are short and conical (Fig 7A), similar in shape to those of both *T*. *adventicia* (Fig 7C) and *T*. *eugeniae* (Fig 7D), however they point slightly inward, appearing to be more convergent than those of all the other species (Fig 7). *Trioza tristaniae* was described as a gall-forming triozid hosted by *Lophostemon confertus* [28], with these two ecological characters clearly distinguishing this species from *T*. *melaleuca* (Table 1). Examination of the only syntype of *Trioza tristaniae* wing preserved at ANIC, enabled the identification of several additional characters that can provide a useful morphological comparison (Fig 4F). The wing of *Trioza tristaniae* is much larger than that of *T*. *melaleucae*, with larger cell cu1 that appears taller than that of *T*. *melaleucae*, due to a longer vein Cu_1b_ and a more pronouncedly arched vein Cu_1a_. In *T*. *tristaniae*, vein Cu is almost the same length as vein Cu_1b_ (Fig 4F), making the value of cell cu1 the lower one across all the Myrtaceae-feeding species examined here (Table 1). On the other hand, in *T*. *melaleucae*, Cu is almost twice as long as Cu_1b_ (Fig 4A). No information was provided by Froggatt in regard to the sex of the psyllid from which the wing was collected. The diagnostic information, however, would retain its value independently of the sex: being 3 mm in length, the wing of *T*. *tristaniae* is significantly longer than that of *T*. *melaleucae*.

Due to the risk of misidentification of *T*. *melaleucae* with the exotic high priority pest *Trioza erytreae*, morphological details are provided to compare the two taxa (Figs 4–7; Table 1). *Trioza melaleucae’s* wings (Fig 4A) are much shorter than those of *T*. *erytreae* (Fig 4E), with the latter showing a value of cell cu1 higher, due to a shorter length of vein m_1+2_. In lateral view, female terminalia of *T*. *erytreae* (Fig 5E) are larger, with both proctiger and subgenital plate being longer than those of *T*. *melaleucae* (Fig 5A). The female proctiger of *T*. *erytreae* is narrowed at about 2/3 of its length, with a post-anal bump, and terminating with a thin point, while that of *T*. *melaleucae* does not have a post-anal bump. In lateral view, the male terminalia of T. erytreae are distinctive, with slender parameres, that are elongated to reach the height of proctiger, with no posteriorly facing tip (Fig 6E), while those of *T*. *melaleucae* are shorter, with a posteriorly facing sclerotized tip (Fig 6A). In lateral view, the male proctiger of *T*. *melaleucae* is round (Fig 6A), while that of *T*. *erytreae* has round lateral wings at the base and becomes thinner in the upper half (Fig 6E). Finally, the genal processes of *T*. *erytreae* are almost as long as the vertex (Fig 7E), while those of *T*. *melaleucae* are about half the vertex length (Fig 7A).

#### 3.1.4. Colouration

*3*.*1*.*4*.*1*. *Adult*. Overall colour of the body consistently different between males and females. Females spanning from a light-brown orange to brown (Figs 1B and 1D and 2A and 2C), males are darker, from dark-brown/dark orange to black (Figs 1A and 1C and 2B and 2D). Independently from this sexual dimorphism, the legs of both sexes tend to a lighter colouration (yellowish) from the tibia down to the claws, while the femur remains of the same colouration as the body. Additionally, both the first and the last segment of the abdomen are white, both in males and females (Figs 1A–1D and 2A–2D). Eyes dark red to burgundy. Genal processes have the same colour as the head, brown for females and dark brown to black for males. Antennae with segments 1 and 3 light orange, 2 and 4 orange at base progressing to dark brown to black terminally; segments 5–8 dark brown to black, unicolorous. (Fig 1E and 1F). Wings hyaline with darker veins tending to light brown and a darker spot on the veins across vein A and cell a1, visible dorsally when the psyllids have folded wings (Fig 1E and 1F). Terminalia brown for both sexes.

*3*.*1*.*4*.*2*. *Nymphs*. Habitus as in Fig 3. Colour of the cephalo-thoracic plate varies from dark yellow/orange to a dark brown. Eyes generally brown tending to red.

#### 3.1.5. Structure

*3*.*1*.*5*.*1*. *Adult*. Habitus as in Figs 1A–1D and 2A–2D. Body broad, compact (Figs 1 and 2). Overall size, from genal process to apex of folded wings, barely reaching 3 mm in the longer females and averaging 2.5 mm for males. Head as in Fig 1G and 1H: vertex elongated, up to 0.75 times as long as wide; genal processes conoid, rounded externally, culminating with a slightly pointed end. Antennae of intermediate length, just 1.2–1.37 times longer than head width. Last antennal segment with two unequal setae. Thorax: mesopraescutum narrower than head, mesoscutum larger but quite short. Head, genal processes and thorax covered in short setae. Fore wings elongated, up to 2.74 times as long as wide, of similar sizes for males and females. Vein Rs shorter than M (0.6–0.9 times), straight for approximately half of its length and then turning upward to the margin of wing. Pterostigma and costal break absent, anal break present and distant from vein Cu_1b_ (Fig 1E and 1F). Tibia with 3+1 spurs. Male terminalia small, proctiger broadly rounded in lateral view without lateral lobes and longer than parameres. Parameres short (0.1 mm), from a lateral point of view pyriform, with dorsolateral lobe, terminating with a darkly-coloured sclerotised apices pointing backward (Fig 1J). Aedeagus short and thin, sinuate in shape (Fig 6A). Subgenital plate, proctiger and parameres covered in setae. Female terminalia short. In lateral view, proctiger without post-anal bump but with a uniform curvature in lateral view that is culminating with a rounded tip. Anal ring relatively long (ratio of proctiger length to circumanal ring only 1.43–1.67). In lateral view, ventral margin of proctiger showing two lobes, about 1/3 of proctiger length (Figs 1I and 5A). Proctiger longer than subgenital plate (up to 1.7 times) (Fig 1I). Ovipositor apex with no serrations above and five reduced serrations below (Fig 5A), valvulae dorsalis slightly convex dorsally.

*3*.*1*.*5*.*2*. *Nymph*. Habitus as in Fig 3. Nymphs are flat, oval in shape, rounded at both extremities, about 1.8 times longer than wide (1.45 mm long and 0.8 mm wide). Margins covered in truncate sectasetae, about 0.01 mm long, arranged contiguously and regularly in a single row, except on the apex of wing pads (Fig 3C), where they are not present. When the filamentous waxy exudate produced by the sectasetae is present, the full length of sectasetae+filament is about 0.03 mm, but size can vary when the filament breaks (Fig 3C and 3E). Antennae short, about half the head width. Circum-anal pore ring in ventral position, about 0.25 mm long and 0.05 mm wide (Fig 3D and 3F).

#### 3.1.6. Measurements

Adult measurements are in mm (4 ♂♂, 4 ♀♀). Length of body (vertex to terminalia) ♂ 1.51–1.65, ♀1.63–1.78; length of body (vertex to apex of folded wings) ♂ 2.47–2.73, ♀ 2.63–3.02; width of head (HW) ♂ 0.49–0.51, ♀ 0.45–0.50; length of genal processes (GCL) ♂ 0.10–0.12, ♀ 0.08–0.11; length of vertex (VL) ♂ 0.18–0.21, ♀ 0.15–0.18; width of vertex (VW) ♂ 0.27–0.30, ♀ 0.26–0.31; length of antenna (AL) ♂ 0.61–0.67, ♀ 0.50–0.62; length of fore wing (WL) ♂ 2.09–2.36, ♀ 1.98–2.35; width of fore wing ♂ 0.72–0.86, ♀ 0.71–0.84; length of vein Rs ♂ 0.93–1.08, ♀ 0.92–1.04; length of vein M(M) ♂ 1.10–1.23, ♀ 1.03–1.29; length of vein M1+2 (M1) ♂ 0.37–0.46, ♀ 0.39–0.47; marginal width of cell m1 ♂ 0.31–0.37, ♀ 0.31–0.40; marginal width of cell cu1 ♂ 0.39–0.43, ♀ 0.35–0.45; length of vein Cu1b ♂ 0.13–0.20, ♀ 0.16–0.22; value of cell cu1 ♂ 2.05–2.67, ♀ 2.19–2.28; value of cell m1 ♂ 1.17–1.31, ♀ 1.09–1.36; length (height) of proctiger (PL) ♂ 0.13–0.16; length of paramere ♂ 0.09–0.10; length of proximal aedeagal segment ♂ 0.12–0.15; length of distal aedeagal segment ♂ 0.08–0.11; length of proctiger (PL) ♀ 0.17–0.22; length of circumanal ring (CL) ♂ 0.11–0.14; length of subgenital plate (SL) ♀ 0.11–0.15.

#### 3.1.7. Ratios

GCL:VL ♂ 0.48–0.63, ♀ 0.5–0.67; VL:VW ♂ 0.6–0.75, ♀ 0.52–0.65; VL:HW ♂ 0.35–0.42, ♀ 0.31–0.38; AL:HW ♂ 1.2–1.37, ♀ 1.06–1.26; PL:HW ♂ 0.25–0.32, ♀ 0.38–0.47; PL:CL ♀ 1.43–1.67; PL:SL ♀ 1.33–1.69; WL:HW ♂ 4.1–4.63, ♀ 4.36–4.8; WL:WW ♂ 2.74–2.91, ♀ 2.71–2.9; Rs:M ♂ 0.83–0.89, ♀ 0.83–0.89; M1:M ♂ 0.33–0.41, ♀ 0.33–0.4.

#### 3.1.8. Etymology

The name *Trioza melaleucae* refers to the host plant *Melaleuca alternifolia*. The case is genitive.

#### 3.1.9. Distribution

The psyllid was collected in the states of Queensland (Dimbulah, Tweed Valley) and New South Wales (Teven, Richmond Valley, Wilson River, Bungawalbin, Grafton and Port Macquarie), in Australia. It is considered endemic to Australia with a distribution from mid-New South Wales to Far-North Queensland, possibly mirroring that of the host plant, which is found in sub-tropical to temperate eastern Australia.

#### 3.1.10. Host plant

**Fig 8:**
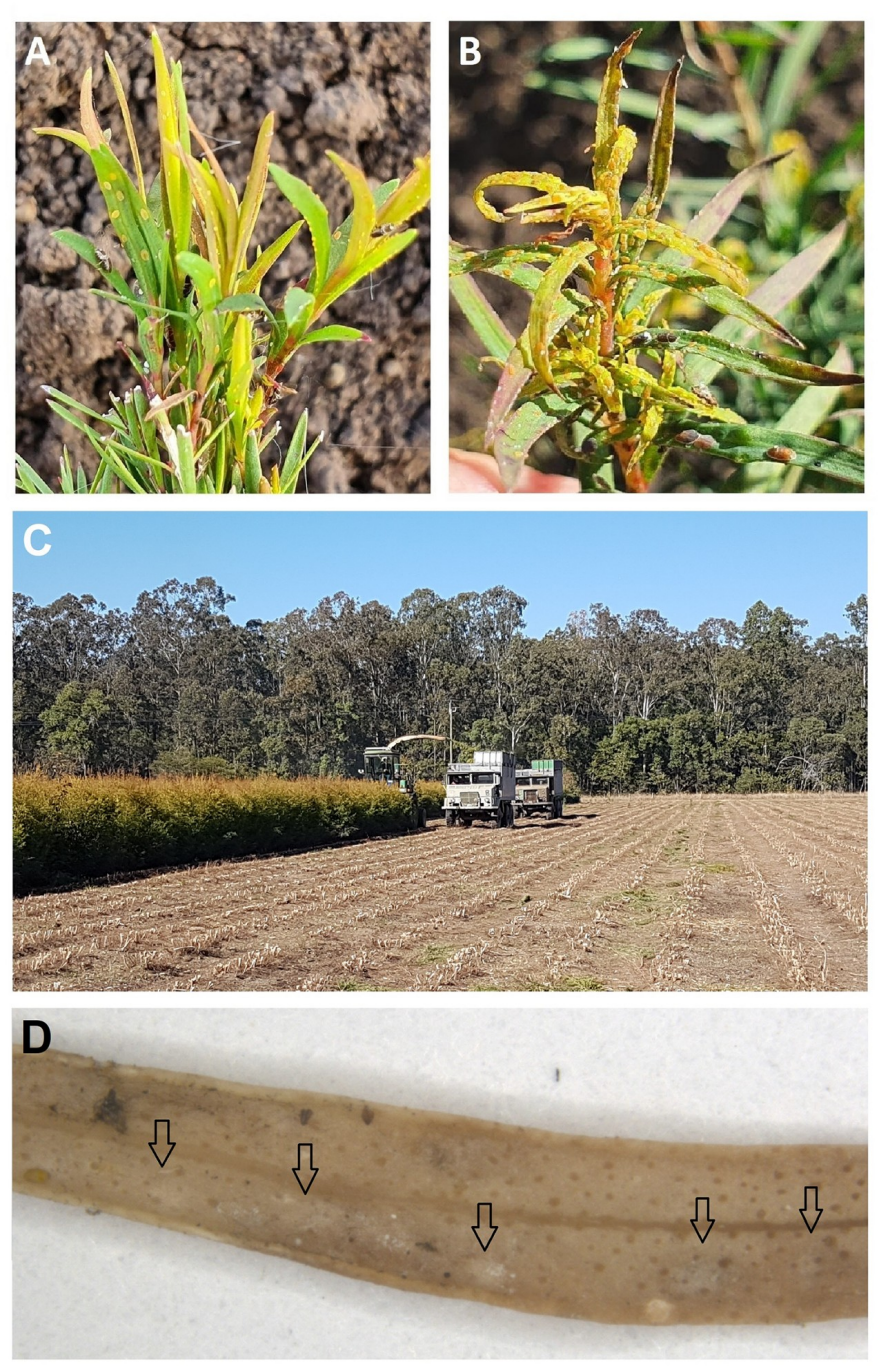
*Melaleuca alternifolia* attacked by *Trioza melaleucae*. The initial infestation with adult psyllids and very young nymphs (**A**) can be compared to the final stages of the infestation, with larger 5^th^ instar nymphs causing leaves curling (**B**). Yearly, *M*. *alternifolia* is harvested (left) removing any biomass present above ground (right) (**C**). Pitting on the leaves are highlighted by arrows (**D**). Photos courtesy of Peter Entwistle and Tony Larkman.

All developmental stages of the psyllid (eggs, nymphs and adults) could be collected from the tea tree, *M*. *alternifolia*, suggesting it is the host plant of *T*. *melaleucae*, as the plant on which the psyllid can complete its life cycle, from eggs to adulthood, in agreement with the most recent definitions of “host plant” for psyllids, by Burckhardt *et al*. [30]. The nymphs of *T*. *melaleucae* were observed feeding on the new growth of *M*. *alternifolia*, causing pitting, leaf curling, discolouration and wilting (Fig 8). Additionally, adult psyllids could be collected from, and observed in great numbers, on *Citrus* plants and various other monocots and dicots in Queensland, from plants of an orchard sharing a boundary with a tea tree plantation (Miles, A. 2021 –personal communication). No nymphs were collected from *Citrus* or other nearby plants, with no leaf damage reported. *Citrus* plants and other uncatalogued taxa therefore can be considered casual hosts for this psyllid [30]. In New South Wales, the plant *Waterhousea floribunda* (F.Muell.) B.Hyland, often located in proximity of tea tree plantations along water ways, has been observed to host *T*. *melaleucae* after the tea tree was harvested (Entwistle, P. 2021 –personal communication). This suggests *Waterhousea floribunda* can be considered an overwintering plant for the psyllid *T*. *melaleucae* [30]. Similarly, in New South Wales, adults of *T*. *melaleucae* were collected from *Melaleuca linariifolia* Sm., while adults and eggs were observed on *Melaleuca ericifolia* Sm. (Entwistle, P. 2021 –personal communication), suggesting both these plants can be casual hosts. Lastly, a collection was made in 2004 from *Calistemon viminalis* (no nymphs or galls seen) (Percy, D. 2021 –personal communication).

#### 3.1.11. Remarks

The nymphs of *Trioza melaleucae* are responsible for the curling of the young leaves of *Melaleuca alternifolia*, which leads to leaf death. In the rare occasion a leaf survives, such as when the plant is sprayed with insecticides, pits can be observed on the leaves. Otherwise, the leaf curling causes leaf death before pitting can appear. Harvest of tea tree occurs annually and removes the entire aerial portion of the plant (usually between 1–6 cm above ground level; Fig 8C). Any remnant vegetation is then slashed off prior to re-coppicing, leaving nothing for this insect to use as a host. This drives *T*. *melaleucae* to nearby plants, including *Citrus*, from which it tends to disperse soon after, disappearing from the local area.

### 3.2. Molecular analysis

**Fig 9:**
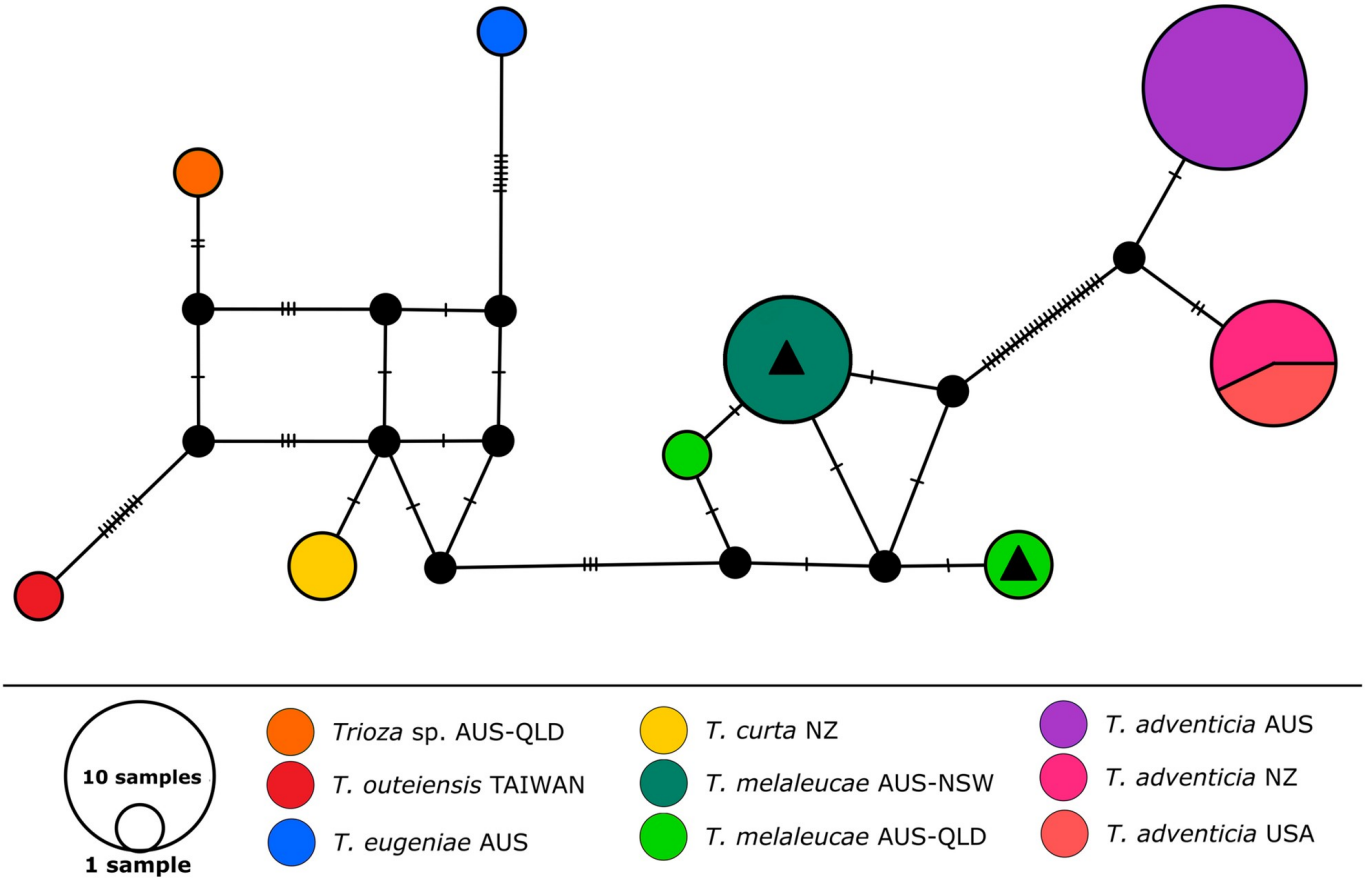
Haplotype network analysis of the samples included in this study, obtained using 33 partial COI DNA sequences. The Queensland (light green) and the New South Wales (dark green) populations of *Trioza melaleucae* are compared to populations of *T*. *adventicia* from New Zealand (fuchsia), USA (pink) and Australia (purple); *T*. *eugeniae* from Australia (blue), *T*. *curta* from New Zealand (yellow), *T*. *outeiensis* from Taiwan (red) and a *Trioza* sp. from Queensland (orange). Each mutation is represented by a hatch mark while the size of the circles corresponds to the number of sequences included. Black triangles represent the sequences generated in this study.

A total of eight partial COI sequences (Accession numbers MW655735-MW655742) were generated in this study and uploaded on GenBank (S1 Table). The pairwise distance matrix performed on the COI sequences obtained in this study recorded a 1–1.3% genetic distance between the populations from Queensland and those from New South Wales. When comparing the sequences obtained here against those publicly available on the GenBank database, a close match (>99%) was discovered with a single COI sequences of a *Trioza* species from Queensland (*Trioza* sp.QLDCal04, accession number MG988861.1, collected in Queensland on *Callistemon viminalis–*Percy, D. 2021, personal communication) included in a phylogenetic work by Percy *et al*. [31], which clearly represents the same species (Fig 9; light green haplotype without black triangle). The second closest match was with two sequences of *T*. *curta* from New Zealand, with > 5%, and *T*. *adventicia*, with > 16%, genetic distance. The COI sequences of *T*. *melaleucae* were added to the dataset which included these species recently presented by Taylor and Martoni [12] to generate the COI haplotype network of Fig 9. In addition, COI sequences for another two Myrtaceae-feeding *Trioza* were included in this analysis: *Trioza outeiensis* Yang from *Syzygium buxifolium* Hook. & Am. (from Taiwan, in red in Fig 9) and a *Trioza* sp. from *Rhodomyrtus* (DC.) Rchb. sp. (from Queensland, Australia, in orange in Fig 9). Both these sequences had previously been grouped together with *Trioza melaleucae* (as “*Trioza* sp.QLD”) showing a close genetic similarity [31]. The haplotype network analysis shows the *T*. *melaleucae* populations from Queensland and New South Wales (Fig 9, in light green and dark green, respectively) clustered together with *T*. *curta* from New Zealand (Fig 9, in yellow) and *T*. *eugeniae* from Australia (Fig 9, in blue). This group is well separated from the *T*. *adventicia* populations from New Zealand, USA and Australia (Fig 9, in fuchsia, pink and purple, respectively).

## 4. Discussion

### 4.1. Ecological and economic impact

The association between *Trioza melaleucae* and tea tree makes this psyllid a potential pest for this horticulturally important plant. Indeed, *T*. *melaleucae*’s nymphs cause leaf curling, wilting and pitting of foliage when feeding on juvenile leaves, which are important for the tea tree industry. When the nymphs are present in large numbers, the tea tree leaves start curling so quickly that often the pits are not seen on the leaves. This is possibly due to the softer consistency of the young *Melaleuca* leaves, as compared to the thicker leaves of *Syzygium* that can show signs of pitting from *T*. *adventicia* before curling [12]. However, shallow pits are present on the leaves if the nymphs are removed before these curl up completely and fall. For these reasons, *Trioza melaleucae* has been considered an important pest for the tea tree industry from ~2017 (previously reported as “*Trioza* sp.”; [13]), forcing growers of the Australian Tea Tree Industry Association (ATTIA) to use petroleum oil insecticides (Permit number: PER82090). The usually strong psyllid-plant association [30] is possibly influenced by the cultivation techniques used for *M*. *alternifolia* that may impact the psyllids’ life cycle. Tea tree plants are harvested yearly by cutting newly grown leaves, where the psyllids nymphs have been predominantly observed to feed and grow to adulthood. The remaining above-ground biomass is then destroyed post-harvest. Thus, the presence of a large amount of new growth, without support of older leaves and stems to maintain the plant generally, allows the insects to colonize and kill all the new foliage. When tea tree is harvested, *T*. *melaleucae* can use other species of *Melaleuca*, as well as other plants, such as *Citrus*, *Waterhousea* and possibly *Calistemon*, to maintain their population until *Melaleuca alternifolia* grows back. This phenomenon has raised alarm among *Citrus* growers in Australia, who might erroneously mistake *T*. *melaleucae* for the exotic pest *T*. *erytreae*, the African Citrus psyllid, a notorious vector of plant pathogens [32]. The description presented here, together with the COI sequences generated, will provide a useful tool to correctly identify *T*. *melaleucae* from *T*. *erytreae* and other *Trioza* species.

The relatively recent record of *T*. *melaleucae* psyllids in outbreak numbers raises the question as to where this psyllid originated. While *Melaleuca alternifolia* is cultivated only in the states of Queensland and New South Wales, *T*. *melaleucae* could have switched host from a different plant. The presence of overwintering plants and food plants reported here may suggest that this psyllid is prone to associations with a variety of hosts. Furthermore, *M*. *alternifolia* is also present in the wild in Queensland and New South Wales and may have hosted *T*. *melaleucae* for a long time, albeit in smaller numbers. The increase of tea tree cultivation during the past 60 years may have contributed to the spread of *T*. *melaleucae* when new plantations were initiated and grown in monoculture.

Another Australian psyllid, *Boreioglycaspis melaleucae* Moore, is hosted by a *Melaleuca* species, *M*. *quinquenervia* (Cav.) S. T. Blake [33]. Similar to the association between *T*. *melaleucae* and *M*. *alternifolia* presented here, the strong psyllid-host plant association can damage the leaves of *M*. *quinquenervia* heavily. However, *M*. *quinquenervia* is considered an invasive species in the Florida Everglades, USA, where *B*. *melaleucae* now has been introduced as a biological control agent [34] and has been shown to be well established and effective [35]. While the two *Melaleuca* host plants are different species, future studies should test the potential survivability of *T*. *melaleucae* on *Melaleuca quinquenervia* to determine if the triozid also could be used as a biological control agent for *M*. *quinquenervia*. The lack of records of *T*. *melaleucae* on *M*. *quinquenervia* may simply be due to the absence of this plant around tea tree plantations, where *Trioza melaleucae* was collected.

### 4.2. The Myrtaceae-feeding *Trioza* spp. of Australia, New Zealand, and Taiwan

With the description of *Trioza melaleucae*, the number of species belonging to this genus in Australia is increased to twelve. However, the genus *Trioza* occurs worldwide and often has been described as a “catch-all” genus [36], being considered a non-monophyletic catch-all genus [3]. The phylogenetic analysis conducted by Percy and colleagues [31], confirmed this, showing strongly supported clades within the family Triozidae and a polyphyletic genus *Trioza*, often including *Trioza* species together with other genera [31]. For example, the European *Trioza remota* was grouped together with species of the genera *Hemitrioza*, *Pariaconus* and *Pauropsylla* in their “group A”, well separated from the Australian *Trioza* species included in their study (*T*. *adventicia* and *T*. *melaleucae*), which were placed in their “group G”. The COI haplotype network obtained here place *Trioza melaleuca* within the Myrtaceae-feeding group of *Trioza* present across Australia (*T*. *eugeniae* and *T*. *adventicia* [12]; and *Trioza* sp. [31]) and New Zealand (*T*. *curta* and *T*. *adventicia* [37]), as well as the species *T*. *outeiensis*, from Taiwan. This is consistent with the information presented in the work of Percy and colleagues [31], who included sequences of *T*. *melaleucae* (as “*Trioza* sp.QLD”) and grouped it together with other Myrtaceae-feeding taxa, including *T*. *adventicia* (as “*T*. *eugeniae*”, in Group “G”). The analysis presented here provides novel information on the relationship between those two species, *T*. *melaleucae* and *T*. *adventicia*, and the two *Trioza* present in Australia and New Zealand: *T*. *curta* and *T*. *eugeniae*. Indeed, we demonstrated that, despite different geographical distributions, *T*. *melaleucae* is more closely related to the New Zealand endemic species *T*. *curta*, supporting the hypothesis that this latter species might be of Australian origin [37]. The COI haplotype network presented here adds novel sequences of *T*. *melaleucae* to a previously presented dataset [12], with the genetic distances confirming the new species described here is congeneric with the other Myrtaceae-feeding species. This consequently confirms that *Trioza eugeniae* and *Trioza curta* are also part of the strongly supported group of Myrtaceae-feeders presented by Percy and colleagues, despite not being included in that study [31]. To fully understand the composition of this Myrtaceae-feeding triozid genus separated from the other *Trioza*, future works should aim to sample a wider number of *Trioza* psyllids, including species from Europe, Australia and, most importantly, the only other described species of Australian *Trioza* hosted by Myrtaceae, the gall-forming *T*. *tristaniae* Froggatt 1903. Unfortunately, this *Lophostemon-*feeding species has not been recorded since 1903, when it was described based on specimens collected near Gympie, Queensland, Australia [28].

Finally, considering the large number of psyllids that are known to vector plant-pathogenic bacteria [38], future research should assess if plant pathogens are involved in the relationship between *Trioza melaleucae* and its host plant, *Melaleuca alternifolia*. Ultimately, the description of *Trioza melaleucae* presented here has led to a better understanding of the relationships across the Myrtaceae-feeding *Trioza* of the region. This highlights the importance of including a description of morphological characters and molecular variation, in addition to providing the ecological context when describing species, as a fundamental step towards a better understanding of our biodiversity.

## Supporting information

S1 Table Accession numbers of all 33 COI sequences used in this study. Sequences in Bold were generated in the present study.
(DOCX)
