# Supplementary material for: Description of an Australian endemic species of Trioza (Hemiptera: Triozidae) pest of the endemic tea tree, Melaleuca alternifolia (Myrtaceae)
Source: PLoS One. 2021 Sep 22;16(9):e0257031. doi: 10.1371/journal.pone.0257031 (PMC8457488; doi:10.1371/journal.pone.0257031)
Supplement: S1 Table — Sequences in Bold were generated in the present study. (DOCX) [file pone.0257031.s001.docx]

**S1 Table:** Accession numbers of all 33 COI sequences used in this study. Sequences in Bold were generated in the present study.

| Species | Origin | Number of sequences | Accession Numbers |
| --- | --- | --- | --- |
| *Trioza adventicia* | Australia | 12 | KY294152; MK716242-MK716252 |
| *Trioza adventicia* | New Zealand | 4 | KY294146; MG132511-MG132513 |
| *Trioza adventicia* | USA | 3 | KY294150-KY294151; KY294153 |
| *Trioza curta* | New Zealand | 2 | MG132537-MG132538 |
| *Trioza eugeniae* | Australia | 1 | MK716253 |
| *Trioza melaleucae* | Australia | 8 | **MW655735- MW655742** |
| *Trioza melaleucae* | Australia | 1 | MG988861 |
| *Trioza outeiensis* | Taiwan | 1 | KY294159 |
| *Trioza* sp. | Australia | 1 | MG988859 |
